# Micro-Structured Polydopamine Films via Pulsed Electrochemical Deposition

**DOI:** 10.3390/nano9020242

**Published:** 2019-02-11

**Authors:** Jing Lin, Sven Daboss, Dominik Blaimer, Christine Kranz

**Affiliations:** Institute of Analytical and Bioanalytical Chemistry, Ulm University, Albert-Einstein-Allee 11, 89081 Ulm, Germany; jing.lin@uni-ulm.de (J.L.); sven.daboss@uni-ulm.de (S.D.); dominik.blaimer@uni-ulm.de (D.B.)

**Keywords:** polydopamine, pulsed deposition, scanning electrochemical microscopy, functional groups, electron transfer

## Abstract

Polydopamine (PDA) films are interesting as smart functional materials, and their controlled structured formation plays a significant role in a wide range of applications ranging from cell adhesion to sensing and catalysis. A pulsed deposition technique is reported for micro-structuring polydopamine films using scanning electrochemical microscopy (SECM) in direct mode. Thereby, precise and reproducible film thicknesses of the deposited spots could be achieved ranging from 5.9 +/− 0.48 nm (1 pulse cycle) to 75.4 nm +/− 2.5 nm for 90 pulse cycles. The obtained morphology is different in comparison to films deposited via cyclic voltammetry or films formed by autooxidation showing a cracked blister-like structure for high pulse cycle numbers. The obtained polydopamine spots were investigated in respect to their electrochemical properties using SECM approach curves. Quantitative kinetic data in dependence of the film thickness, the substrate potential, and the used redox species were obtained.

## 1. Introduction

Polydopamine has interesting chemical and physical properties, which renders this functional polymer as a versatile material for a multitude of applications ranging from biomedical to energy-relevant applications in battery research, as protection films in corrosion, and as coatings for adsorption purposes [1,2,3,4,5]. Inspired by mussel adhesive proteins, Lee et al. first reported PDA as coating material in 2007 [6]. Mussel adhesive proteins have a high content of catechol (3,4-dihydroxybenzene) due to the presence of 3,4-dihydroxy-l-phenylalanine (DOPA) and high content of primary and secondary amines due to lysine (Lys) and histidine residues. In this first report, PDA films were obtained by dip coating from basic (pH = 8.5) dopamine solutions. Since then, PDA has been recognized as universal coating material adhering to almost any material including teflon and on any geometry (nanoparticles, porous substrates, etc.) [7,8,9]. The presence of functional groups such as catechol, amine and imine groups, and its high biocompatibility renders PDA an ideal material for biomedical applications [1], sensing applications (e.g., for bioaffinity sensing [10], DNA sensing [11], for intracellular pH sensing [12]), and as substrate for cell adhesion [13]. Deposition of PDA films can be achieved via a simple one step dipping procedure in slightly basic aqueous dopamine solutions (>pH 7.4) through oxidation and subsequent self-polymerization in the presence of oxygen [6] or other oxidants such as reactive oxygen species (ROS) generated via UV radiation [14] or Cu^2+^/H_2_O_2_ [14] in phosphate buffered, TRIS buffered or hydrogencarbonate-buffered solutions. Among other factors, the obtained film thickness varies predominantly with the dipping times, the concentration of dopamine and the used buffered solution [15].

**Scheme 1:**
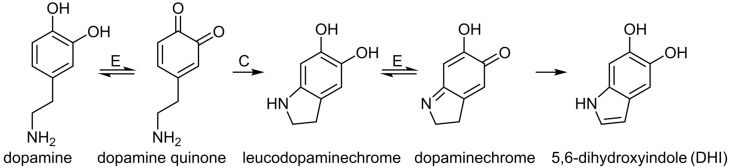
First electrochemical steps for the formation of polydopamine. The oxidation of dopamine is generally agreed on is an ECE mechanism leading to the formation of dopaminechrome, which further leads to the formation of 5,6-dihydroyindole. 5,6-dihydroyindole leads through further oxidation and reactions to the formation to polydopamine.

Polymer films derived from catecholamines as redox active molecules can also be obtained by electrochemically induced deposition. First reports using electrochemical methods to deposit PDA films have been reported by He et al. [3] and by Liu et al. [16]. So far, predominantly cyclic voltammetry has been used to deposit PDA films from deoxygenated solution within a potential sweep range from −0.6 V or −0.5 V to 0.5 V at slightly basic pH values in buffered solution [3,11,16,17,18]. It is reported that electrochemical deposition via cyclic voltammetry results in higher deposition rates and continuous, smooth films at the electrode surface in comparison to film deposition by dipping [17]. As first steps, an electrochemical homogenous coupled chemical–electrochemical (ECE) mechanism forms dopamine–quinone that undergoes intramolecular cyclization to leucodopaminechrome, and further oxidation to dopaminechrome as shown in Scheme 1. Via a subsequent isomerization step, 5,6-dihydroxyindole (DHI) is obtained, and by further oxidation 5,6-indolequinone is formed, which undergoes coupling and further oxidation reactions resulting in an insoluble precipitation at the electrode surface. The level of polymerization and the resulting structure, which is dependent on preparation conditions [19,20,21,22,23] is still under debate, as recently reported by D’Ischia et al. [24]. Structures like supramolecular aggregation of monomers that are held together through charge transfer, π–π stacking and hydrogen bonding, trimeric assemblies along with covalent bonding between units and non-covalent self-assembling of monomers or formed oligomers such as aromatic rings stacking are discussed for the formation of PDA. 

There is an increasing number of applications, which typically require uniform coatings with well controlled thickness and low surface roughness. Frequently, the so far applied deposition routines result in the formation of additional particulate PDA structures at the sample surface or inhomogeneous films [25,26]. Within this contribution, we investigate a pulse deposition regime for electrodeposition of PDA spots employing scanning electrochemical microscopy for microstructuring PDA films, which enables screening experiments in respect to the relation of pulse cycle number with film thickness and surface morphology. 

**Figure 1:**
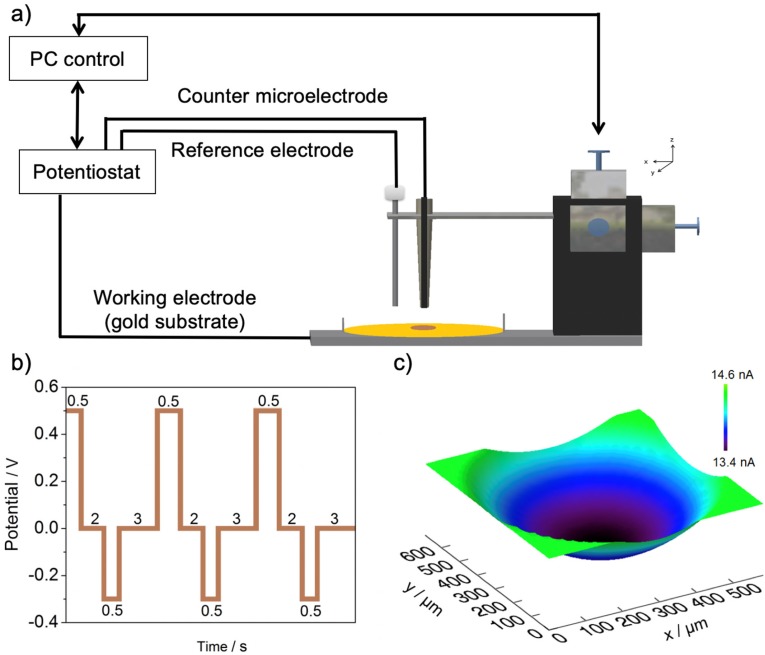
(**a**) Scheme of the SECM direct mode approach for micro-structured PDA deposition. (**b**) Potential pulse profile for electropolymerization. (**c**) 3D SECM image of PDA microspot deposited with 30 cycles. The image is obtained in 5 mM ferrocenedimethanol/0.1 M KCl with a Pt UME (12.5 µm radius); scan velocity 10 µm/s.

Scanning electrochemical microscopy [27] is a versatile electrochemical scanning probe technique, which is widely used for high-resolution studies of interface processes, in catalysis, energy-related topics, corrosion, biomedical research [28], as electroanalytical tool for detection of redox active analytes [29], and to quantitatively determine heterogeneous and homogenous electron transfer rates at various substrates [30]. The microelectrode used as SECM tip is positioned by recording the current at the biased SECM tip in solution containing a redox species with fast electron transfer characteristic while approaching in z-direction the sample surface. These current versus z distance curves are termed approach curves. Such current-distance curves can be fitted by analytical approximations to obtain electron transfer kinetic data [31,32]. For example, SECM has been recently employed to investigate the fouling of electrode surfaces due to the formation of polydopamine during DA detection [33]. SECM is also a suitable tool for in-situ localized surface modifications based on etching and deposition processes forming microscopic and nanoscopic metal structures, conductive polymer and insulators structures [34]. Such surface modifications are usually achieved in feedback mode [35] or direct mode SECM [36]. In direct mode SECM, the SECM tip is used as counter electrode and the substrate as the working electrode. If a potential is applied, the electrical field is defined in dependence of the distance of the SECM tip to the substrate and the size of the electroactive area of the nano- or micro-sized SECM tip, leading to localized modifications as shown in Figure 1a. Pulsed deposition in combination with SECM has been proven to be a versatile method to microstructure polymers and metals typically with dimensions of the used microelectrode. A significant advantage of SECM is that the obtained structures can subsequently be investigated in respect to their electrochemical properties.

Within this contribution, we evaluate the micro-structured deposition of polydopamine using the direct mode of SECM. In contrast to the conventionally applied electrochemical deposition via cyclic voltammetry, pulsed deposition enabled superior control of the achieved film thickness of the obtained polymer spots. To the best of our knowledge, pulse deposition in respect to PDA formation has only be reported for layered PDA hydroxyapatite nanoparticles films [33]. The deposits are also investigated in respect to morphology using atomic force microscopy (AFM), electron transfer characteristics via SECM, and surface functionality using infrared microspectroscopy. 

## 2. Materials and Methods 

### 2.1. Reagents

Dopamine hydrochloride was purchased from Sigma-Aldrich, (Steinheim, Germany). Dopamine was dissolved in 10 mM PBS buffer (pH 7.4) and purged with argon for 20 min before its use. Sodium hydrogen phosphate (Na_2_HPO_4_), sodium dihydrogen phosphate (NaH_2_PO_4_), sodium chloride (NaCl), potassium chloride (KCl), hexaammineruthenium(iii)chloride ([Ru(NH_3_)_6_]Cl_3_) were purchased from Merck, (Darmstadt, Germany). Potassium ferrocyanide was purchased from Fluka, (Buchs, Switzerland). All the solutions were prepared with high purity water (18.2 MΩ cm, Elga Labwater; VWS Deutschland, Celle, Germany). Silicon wafers coated with 5 nm Ti adhesion layer and 95 nm Au were used as substrates for PDA depositions. Prior to use, the Au-coated Si wafer was cleaned in acetone, absolute ethanol and high purity water. 

Pt ultramicroelectrodes (UMEs) was prepared by sealing Pt microwires (12.5 µm radius, Goodfellow, Bad Nauheim, Germany) in borosilicate glass (glass capillaries were purchased from Hilgenberg, Malsfeld, Germany). In order to achieve RG values (ratio of radius of the insulating sheath and the electrode radius) of the microelectrodes of approximately 5, UMEs were pulled using a pipette puller (Narishige PC-10, Tokyo, Japan). Disc-shaped microelectrodes were exposed by grinding and polishing on diamond lapping films (Allied High-Tech Products, Rancho Dominguez, CA, USA) and alumina oxide suspension (particle size: 0.05 µm, purchased from Buehler, Düsseldorf, Germany) on red Technotron cloth (LECO, St. Joseph, MO, USA), respectively. A detailed fabrication protocol is given elsewhere [37]. The fabricated UMEs were characterized by cyclic voltammetry in 5 mM [Ru(NH_3_)_6_]Cl_3_/0.1 M KCl. 

### 2.2. Electrochemical Measurements

A three-electrode set-up was used for characterization of the ultramicroelectrodes with a Pt counter electrode (CE), and an Ag/AgCl (3 M KCl) reference electrode. The experiments were controlled by a CHI660A potentiostat (CH Instruments (Austin, TX, USA). Deposition of polydopamine was performed in direct mode of SECM as illustrated in Figure 1 using a home-built SECM system (software: G. Wittstock, University Oldenburg, Oldenburg, Germany). The UME was positioned at a distance of 160 μm recording an approach curve in 5 mM ferrocenedimethanol/0.1 M KCl and consecutive retracting of the UME to the desired distance after approach. Polydopamine spots were deposited from freshly-prepared, deoxygenated dopamine solution containing 1 mg mL^−1^ dopamine hydrochloride in 0.01 M PBS (pH 7.4), using a potential pulse sequence of 0.5 V/0.5 s; 0.0 V/2 s; −0.3 V/0.5 s; 0.0 V/3 s with varying numbers of applied pulse cycles (1, 3, 15, 30, 60, 90 pulse cycles). The SECM image as shown in Figure 1c was obtained in feedback mode in 5 mM [Fe(CN)_6_]^4−^/0.1 M KCl at a polydopamine spot deposited with 30 pulse cycles. Approach curves at spots with different thicknesses were recorded either in 5 mM [Ru(NH_3_)_6_]Cl_3_ or 5 mM K_4_[Fe(CN)_6_] solution. Kappa (κ) values were determined at different substrate potentials (substrate either biased at −0.1 V or +0.5 V vs. Ag/AgCl quasi reference electrode). Evaluation of the data were performed with Mira software (purchased from G. Wittstock, University Oldenburg, Oldenburg, Germany).

### 2.3. AFM Measurements

All AFM measurements were performed with a 5500 AFM/SPM microscope (Keysight Technologies, Tempe, AZ, USA) with silicon nitride probes (OMCL-TR800PSA, Olympus Corporation, Japan, k = 0.15 N/m) in contact mode either in air or in buffered solution (PBS pH 7.4). The samples used for imaging in air were after the deposition rinsed with ultrapure water and dried in an argon stream. Images were recorded with a scan speed of 0.85 ln/s. Data were analyzed with the Picoview analysis software (Keysight Technologies, Tempe, AZ, USA).

### 2.4. SEM Imaging

Scanning electron microscopy (SEM) images were obtained with a dual beam system, FIB/SEM FEI Helios Nanolab 600, (ThermoFisher Scientific, Eindhoven, the Netherlands) with a through-the-lens (TLD) detector in immersion mode.

### 2.5. IR Microspectroscopy

IR data were obtained in reflection mode using a Spero QCL System (Spero-QT microscope, Daylight Solutions, San Diego, CA, USA) equipped with a 480 × 480 pixel array-based imaging detector using a 480 × 480 pixel array-based focusing objective with a pixel size of 1.4 × 1.4 μm. As light source a broadly tunable mid-infrared quantum cascade laser tunable in the range of 988–1800 cm^−1^ with a spectral resolution of 2 cm^−1^ was used. Spectra were obtained from the bare gold substrate and from spots deposit with 30 pulse cycles, which were polarized at −0.1 v or 0.5 V for 5 min respectively prior to the measurements.

## 3. Results and Discussions

### 3.1. Optimization of the Deposition Process

The advantages of pulsed deposition in direct mode SECM has been described in early experiments for the deposition of, i.e., conductive polymers [38]. The pulse profile was optimized in respect to the uniformity of the obtained spots and achievable film thickness for depositions from a solution containing 1 mg dopamine/mL. At a distance of 160 µm between UME and sample surface, a pulse sequence of 0.5 V/0.5 s; 0.0 V/2 s, −0.3 V/0.5 s and 0.0 V/0.3 s was determined to achieve films with uniform appearance with average diameters of approximately 400 µm, which reflects the dimension of the microelectrode (diameter of electroactive wire and glass surrounding). The first pulse leads to the oxidation of dopamine to dopaminequinone and sufficient long resting times at 0.0 V after the pulse are required to allow that monomers diffuse into the gap between the counter microelectrode and the working electrode and for rearrangement of the formed species. At −0.3 V, dopaminechrome is formed which reacts further as indicated in Scheme 1. At shorter resting potential times, we observed a non-homogenous distribution of the polymer with a non-coated center of the deposited spot. Also, at distances <80 µm we observed that a polymer ring rather than a spot was formed. Hence, all depositions were obtained at a distance of 160 µm and with counter microelectrodes with RG value of 5 to ensure compatibility of the deposited films. In dependence of the number of pulse cycles, a uniform spot can be obtained even at very low cycle numbers such as a single pulse. 

Figure 1c shows a 3D SECM image recorded at a PDA microspot deposited with 30 repetitive pulse cycles using a 12.5 µm (radius) Pt microelectrode as counter electrode. The interest here was not generating particularly small spots rather than using SECM direct mode to generate spots at the sample surface with varying thicknesses, which then could be screened in a subsequent step in terms of their electrochemical properties. Therefore, the UME microelectrode was switched to the working electrode with the sample unbiased; the solution was exchanged to 5 mM ferrocenedimethanol/0.1 M KCl and the microelectrode was positioned at a height of 40 µm prior to recording the SECM images. It is clearly evident in Figure 1c that a uniform symmetric spot was formed with 30 cycles. The PDA spot blocks the electron transfer resulting in negative feedback current compared to the bare gold electrode.

### 3.2. Film Morphology

**Figure 2:**
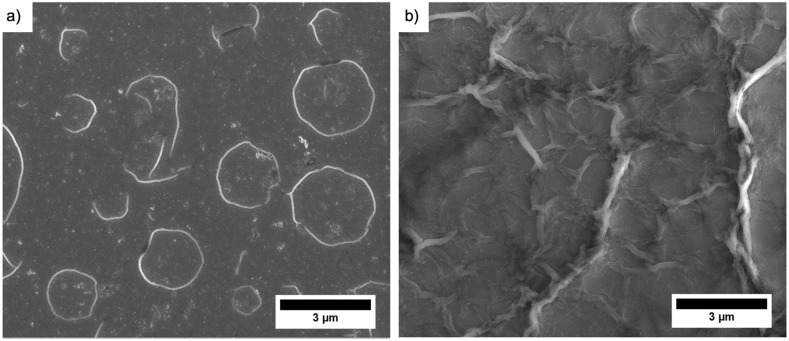
Comparison of film morphology in dependence of electrochemical deposition technique. (**a**) SEM image of a PDA spot deposited via pulse deposition with 10 pulse cycles; (**b**) PDA spot deposited via cyclic voltammetry ten cycles, potential range −0.5 to 0.5 V vs. Ag/AgCl, scan rate 20 mV/s.

For potential applications of PDA as redox active films, the film thickness, but also the film morphology, plays a significant role. AFM and SEM studies have been performed to investigate the film morphology and to determine the film thickness in dependence of the applied number of pulse cycles. The SEM images shown in Figure 2 clearly reveal the quite different film morphology in dependence of the electrochemical deposition technique at otherwise same experimental conditions. Both films were deposited by applying 10 cycles. Figure 2a shows the film deposited via pulsed electrochemical deposition and Figure 2b via cyclic voltammetry. The morphology of the pulse-deposited film appears smooth with circular features that looks like “cracked blisters”, whereas the film deposited via CV results in a more “folded” rough structure with less defined features and higher surface roughness. 

**Figure 3:**
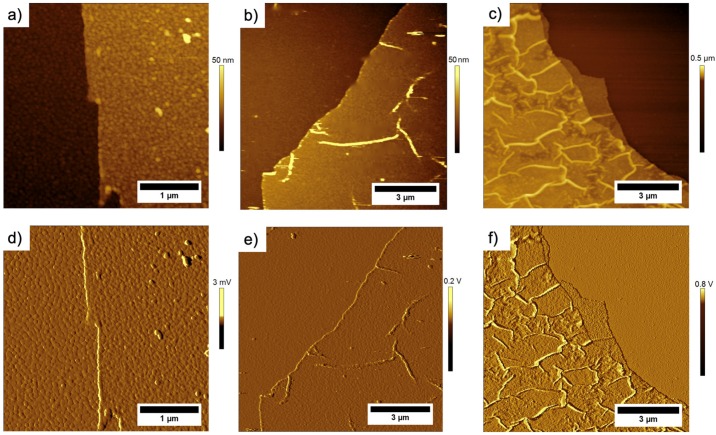
AFM contact mode images of PDA film deposited with different number of pulse cycles. Topography (**a**–**c**) and deflection images (**d**–**f**)) are shown: (**a**,**d**) PDA film deposited with 1 pulse cycle; (**b**,**e**) PDA film deposited with 3 pulse cycles and (**c**,**f**) PDA film deposited with 30 pulse cycles. Images were recorded in contact mode. The displayed images are tilt corrected.

In a next step, the film morphology and surface roughness were determined via AFM. Figure 3 shows exemplary AFM topography and deflection images for different numbers of pulse cycles (1, 3 and 30 pulse cycles). These AFM images were recorded in air. Images were also recorded in buffered solution (see Figure S1) to ensure that drying of the films does not alter the height (i.e., due to possible swelling effects). As the film height and morphology are not altered, the determination of roughness parameter and film thickness was performed in contact mode in air. In general, AFM characterization for chemically or electrochemically deposited PDA films is usually reported for rather small areas (e.g., 1 µm × 1 µm) or AFM images with insufficient quality are presented, which makes the evaluation of the film homogeneity difficult [15,39]. 

**Figure 4:**
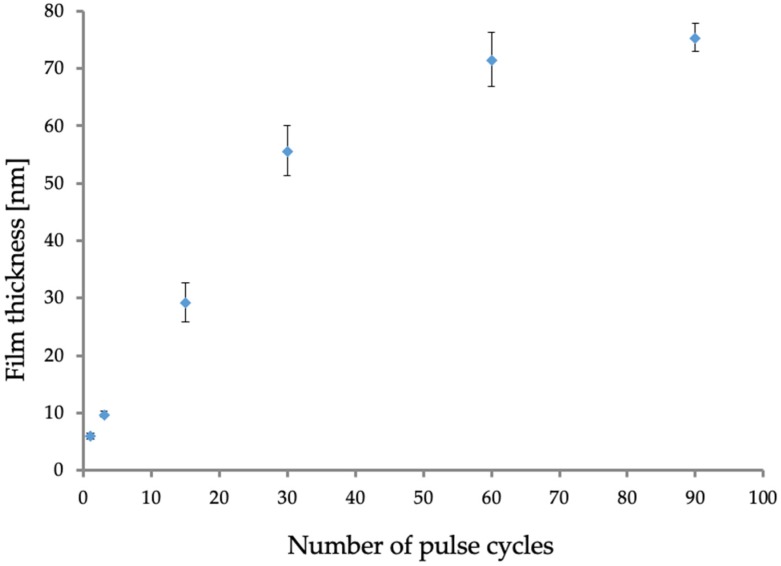
Thickness of the PDA films in dependence of number of pulse cycles. The error bars correspond to ±1 standard deviation over 5 thickness measurements.

As shown in Figure 3a,d, with 1 pulse cycle already a uniform coating of the gold substrate was obtained, with a surface roughness (Sa) of 1.74 nm +/−0.09 and film thickness of 5.9 +/−0.50 nm (N = 5). In comparison to dip-coating, much longer times are required to form a uniform coating. If only one pulse cycle is applied, no “cracked blister-like” features were observed. It should be noted that, for all thickness measurements, such elevated features were avoided. The surface roughness at spots without such features is approximately the same, the film thickness increased to 9.75 +/−0.48 nm (N = 6) for 3 pulse cycles. The film thickness increased with the applied number of pulses (see Figure 4) from 29.27 +/−3.45 nm for 15 pulse cycles to 55.68 +/−4.41 nm for 30 pulse cycles to 70.42 +/−4.12 nm for 60 pulse cycles and then reached a plateau at 90 pulses where an averages thickness of 75.4 nm +/−2.5 nm (N = 6) and a film roughness of 6.54 +/−0.52 (measurements were obtained in blister-free areas) was obtained. With the film thickness, the number of the “circular cracked blisters” increased as shown in Figure 3. It appears that the film deposition happens in layers (also clearly visible for the film deposited with 30 cycles (Figure 3c)). If the surface roughness parameter is determined over larger areas containing these cracked blisters, the roughness parameter Sa increases significantly from 1.74 nm (1 pulse) to 49.5 nm for a film deposited with 60 pulses. The film height displayed in Figure 4 was determined in areas without the cracked blister features.

Interestingly, already a sequence of three pulses leads to a film morphology (Figure 3b), which is characterized by these blister-like features, although only a view of them were observed. These features increase in height, density and size with the number of applied pulse cycles (see Supporting Information Figure S2). We associate these features with hydrogen evolution during the polymerization reaction that may be entrapped in the film, forming blisters. It is clearly evident that due to the electrically insulating properties of the deposited PDA films, further film growth is inhibited, however, the film thickness significantly vary with experimental conditions such as dopamine concentration, pH value, and numbers of deposition cycles (CVs) [11,16,40]. 

**Figure 5:**
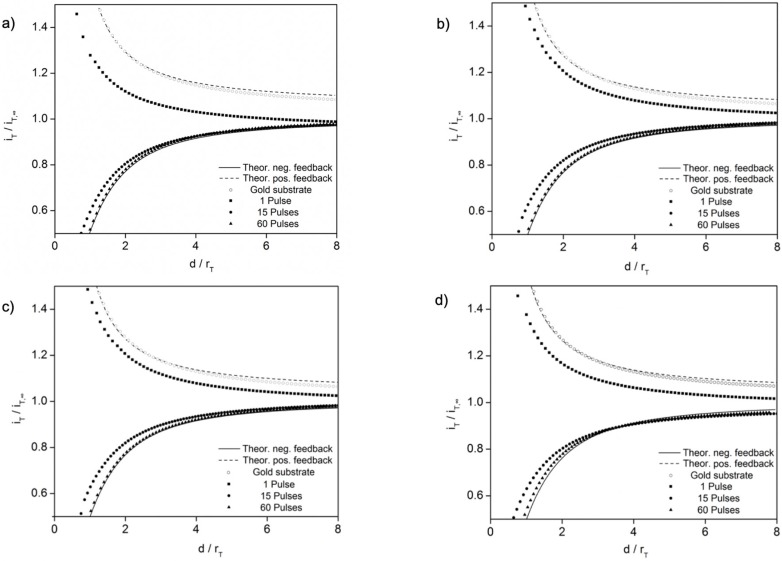
Approach curves recorded at PDA microspots deposited with different number of pulse cycles, in dependence of the applied substrate potential and redox active species. (**a**) Curves recorded in 5 mM [Fe(CN)_6_]^4−^ in 0.1 M KCl, E_substrate_: −0.1 V; E_T_: 0.4 V. (**b**) In 5 mM [Fe(CN)_6_]^4−^/0.1 M KCl, E_substrate_: +0.5 V; E_T_: 0.4 V. (**c**) Curves recorded in 5 mM [Ru(NH_3_)_6_]^3+^/0.1 M KCl, E_substrate_: −0.1 V; E_T_: −0.4 V; (**d**) in 5 mM [Ru(NH_3_)_6_]^3+^/0.1 M KCl, E_substrate_: +0.5 V; E_T_: −0.4 V. All potentials are reported vs. Ag/AgCl and all approach curves were recorded with a scan velocity 1 µm/s.

The electrochemical properties of the deposited films were investigated via SECM. PDA films are characterized by their multitude of functional groups, such as amphiprotic groups (e.g., amino groups), and redox active groups such as hydroquinone and quinone groups. By applying a sufficiently positive bias to the sample, the hydroquinone groups can be oxidized to quinone, which should alter the charge transfer behavior for negatively charged redox mediator in dependence of the film thickness. SECM approach curves were used to investigate the electron transfer kinetics in dependence of the film thickness, the applied substrate potential and used redox active species. Approach curves are plotted as the normalized current i = i/i_inf_ versus the normalized distance L = d/r, where i is the recorded tip current in dependence of the distance d, and i_inf_ is the steady-state current when the tip is far away from the sample surface. SECM approach curves were recorded at different substrate potentials with the sample biased at −0.1 V and at 0.5 V vs. Ag/AgCl, respectively. While the PDA structure is still under debate, it is generally assumed that *o*-quinone and *o*-hydroquinone subunits with their semi-oxidized/semi-reduced forms are present [41]. Hence, by applying a sufficient positive or negative bias to the electrode, the redox groups can be switched from oxidized to reduced state or vice versa. For fast electron transfer and a diffusion controlled process, SECM approach curves recorded at a pure insulator or pure conductor leads to a tip current, which reflects a negative feedback current (i_T_ < i_inf_) in case the diffusion towards the SECM is hindered by the presence of the sample or to positive feedback current (i_T_ > i_inf_) in case of a conducting sample, where the redox species is regenerated, as shown in Figure 5 (theoretical approach curves). In case of finite electron transfer kinetics at modified electrode surfaces [42,43] such as the PDA spots, quantitative kinetic data can be derived from the SECM approach curves. All experimental data presented in Figure 5 are fitted by Lefrou’s approach [32]. The dimensionless rate constant κ = k_eff_ r/D is evaluated with k_eff_ reflecting the constant of the apparent heterogeneous electron transfer rate, D the diffusion coefficient of the used redox mediator and r the active radius of the microelectrode. Approach curves were recorded for a positively charged redox species ([Ru(NH_3_)_6_^3+^]) and a negatively charged redox species ([Fe(CN)_6_]^4−^. The results are summarized in Figure 5 and Table 1. In dependence of the applied substrate potential, it was expected that the oxidation state of the PDA groups (quinone/hydroquinone) influences the electron transfer behavior for the differently charged redox mediators, as electrostatic repulsion may be effective. 

It is clearly evident that deposition with 60 pulses leads to a hindered electron transfer independent of the applied substrate potential and used redox mediators, which almost reflects that of a pure insulator (solid line: theoretical approach curve for an insulator). For 15 pulse cycles the dimensionless rate constant dropped by two orders of magnitude in comparison to the bare gold electrode. For the negatively charged ferrocyanide, kappa is half the value at a negative substrate bias (−0.1 V) with reduced hydroquinone groups compared to a substrate bias of 0.5 V where it is assumed that the oxidized quinone groups are present. The difference in the kinetic data may be explained due to electrostatic repulsion of the negatively charged redox mediator at −0.1 V substrate potential. For the positively charged [Ru(NH_3_)_6_]^3+^ at the negative substrate potential electrostatic attraction should lead to an increased dimensionless rate constant (κ = 0.1617), which is clearly evident compared to the positive substrate potential, where a lower κ value of 0.0617 was obtained. For 1 pulse cycle, a positive feedback current was observed for both redox mediators and oxidized and reduced states of the hydroquinone/quinone couple, whereby the substrate potential again had an influence on the obtained kinetic data following the same trend as observed for 15 pulses. The κ value is highest for the positively charged redox mediator at the negative sample potential assuming that electrostatic interaction (attraction) facilitates the electron transfer, whereas the κ value is lowest for the negatively charged redox species at the negative substrate potential (see Table 1). For only one cycle defects or pinholes in the thin polymer film may also contribute to the observed higher rate constants.

IR microspectroscopy in reflection mode was performed to confirm the presence of hydroquinone or quinone structures in dependence of the oxidation state. Several IR studies of PDA films in dependence of the deposition mode, deposition time and substrate have been reported [44,45]. Figure S3 shows the reflection mode scans of the background (gold electrode) and an oxidized and reduced film of a PDA spot deposited with 30 pulse cycles. Characteristic features associated with functional groups at 1250 cm^−1^ (C-OH stretching), at 1652 cm^−1^ (C=N stretching imine/oxime or N-H bending), and the band at 1736 cm^−1^ associated with the quinone C=O group were evaluated. In dependence of the applied potential, a significant change of the band at 1736 cm^−1^ was observed, which indicates the presence of oxidized quinone groups. 

## 4. Conclusions

We successfully demonstrated a pulsed deposition method for the localized precipitation of polydopamine spots using direct mode SECM. The number of pulse cycles allows a precise control of the PDA film thickness. Interestingly, in comparison to cyclic voltammetric-induced depositions, we obtained a substantially different morphology of the deposited films. The film morphology and thickness were determined by AFM measurements. SECM has the unique advantage that not only surface modifications can be obtained, but the deposited films can also be characterized in respect to their electrochemical properties. Recording SECM approach curves reveals charge transfer kinetics at the deposited spots in dependence of the film thickness and the applied substrate potential. The electron transfer is already significantly hindered at films deposited with 15 cycles. 

## Figures and Tables

**Table 1:** Dimensionless rate constant κ in dependence of substrate potential and redox species.

Number of Pulse Cycles	Substrate Potential vs. Ag/AgCl Redox Mediator: [Fe(CN)_6_]^4−^		Substrate Potential mV vs. Ag/AgCl Redox Mediator: Ru(NH_3_)_6_^3+^	
	−0.1 V	0.5 V	−0.1 V	0.5 V
1	1.4331 ± 0.0402	2.3090 ± 0.0334	2.7222 ± 0.0211	1.6197 ± 0.0274
15	0.0257 ± 0.0034	0.0478 ± 0.0027	0.1617 ± 0.0133	0.0617 ± 0.0082
60	0.0009 ± 0.0003	0.0012 ± 0.0002	0.0068 ± 0.0012	0.0045 ± 0.0007

## Supplementary Materials

The following are available online at https://www.mdpi.com/2079-4991/9/2/242/s1, Figure S1: AFM images comparing air und liquid measurements of PDA spots. Figure S2: SEM images of PDA spots deposited with 10 and 60 pulse cycles, respectively. Figure S3: IR microspectroscopy data of oxidized and reduced PDA spots.
