# Supplementary material for: Micro-Structured Polydopamine Films via Pulsed Electrochemical Deposition
Source: Nanomaterials (Basel). 2019 Feb 11;9(2):242. doi: 10.3390/nano9020242 (PMC6409672; doi:10.3390/nano9020242)
Supplement: Supplementary file 1 [file nanomaterials-09-00242-s001.pdf]

Supporting Information:

Micro-structured Polydopamine Films via Pulsed Electrochemical Deposition

Jing Lin, Sven Daboss, Dominik Blaimer and Christine Kranz*

Institute of Analytical and Bioanalytical Chemistry, Ulm University, Albert-Einstein-Allee 11, 89081 Ulm, Germany.

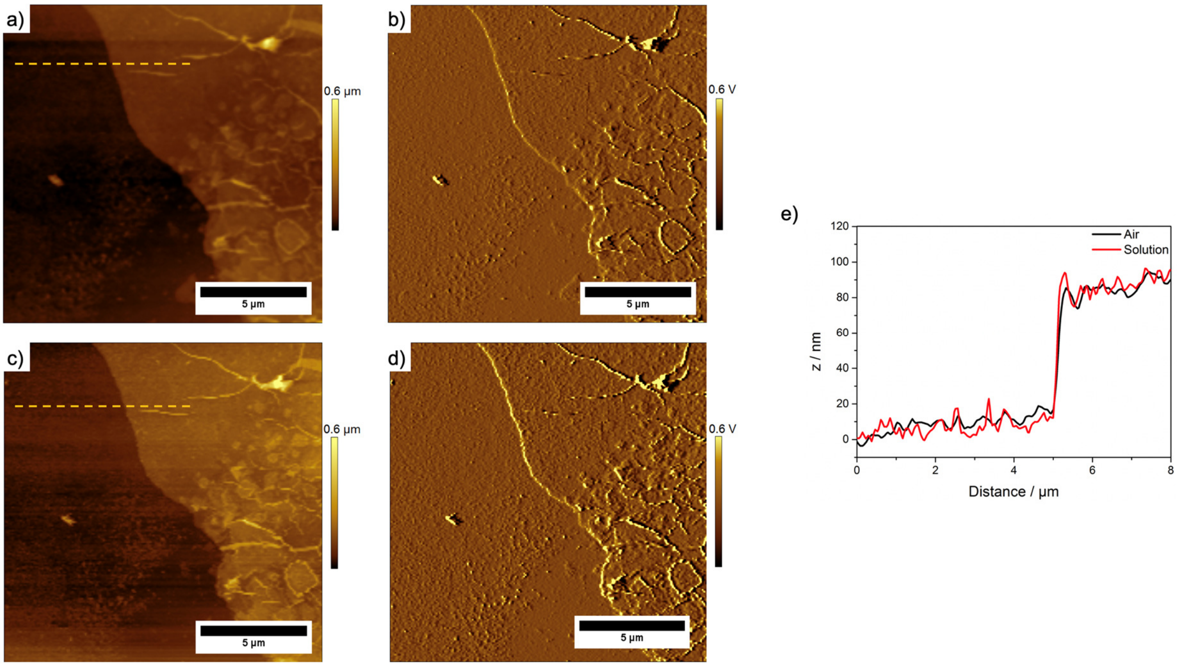

Figure S1. Comparison of AFM topography (a, c) and deflection images (b, d) images of a micro-structured PDA film deposited with 30 pulse cycles. a) Topography in air, b) deflection image in air, c) topography in solution, d) deflection image in solution, e) overlay of height profiles indicated in a) and c) by the dotted line.

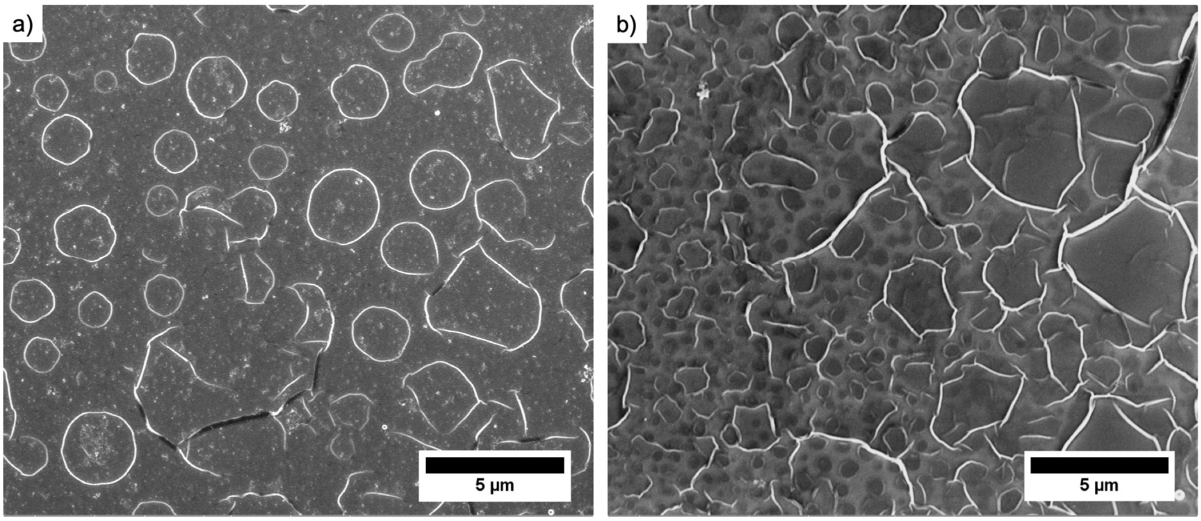

Figure S2. SEM images of PDA spots obtained with a) 10 pulse cycles and b) 60 pulse cycles.

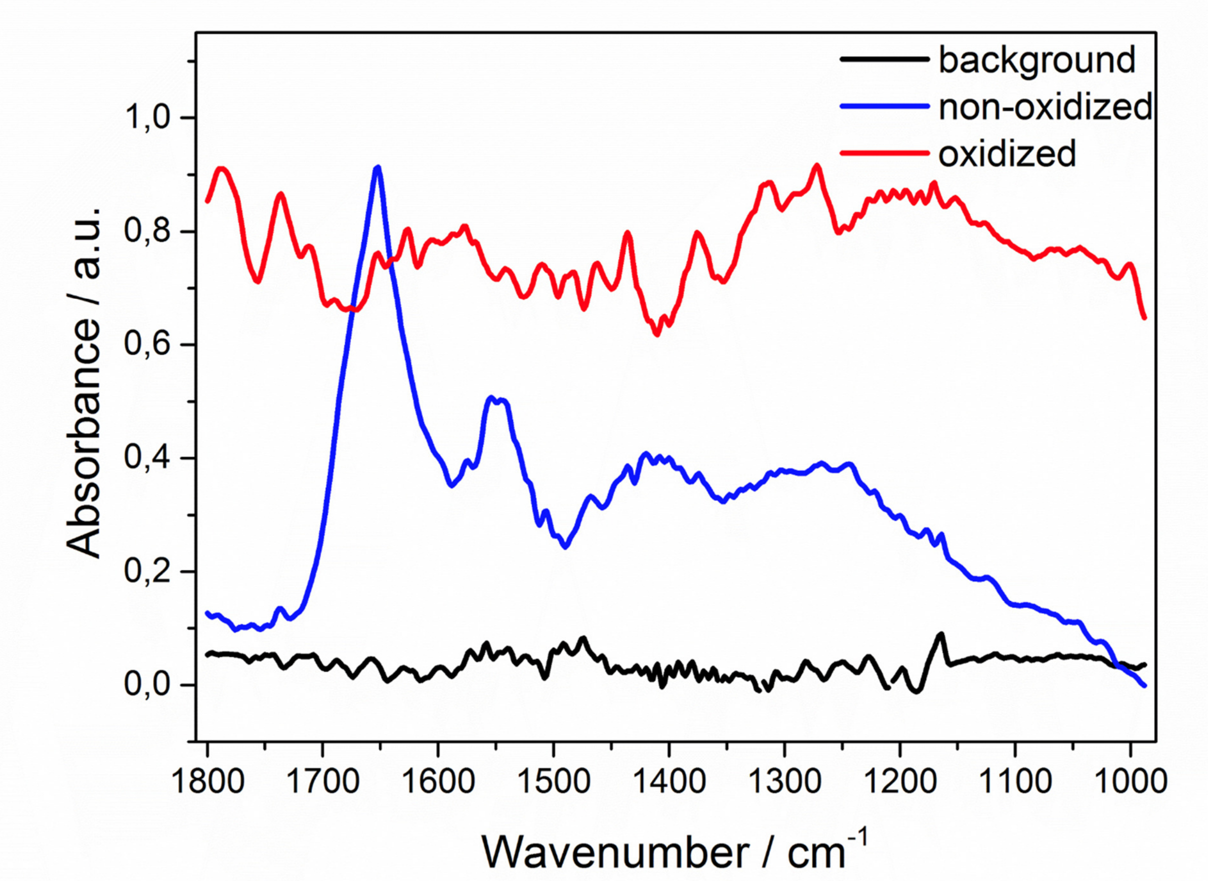

Figure S3. IR reflection spectra of the bare gold electrode (black), the oxidized PDS film polarized for 5 minutes at 0.5 V and the reduced PDA film polarized for 5 min at -0.1 V vs Ag/AgCl.
